# Zinc-mediated activation of CREB pathway in proliferation of pulmonary artery smooth muscle cells in pulmonary hypertension

**DOI:** 10.1186/s12964-021-00779-y

**Published:** 2021-10-11

**Authors:** Genfa Xiao, Guili Lian, Tingjun Wang, Weixiao Chen, Wei Zhuang, Li Luo, Huajun Wang, Liangdi Xie

**Affiliations:** 1 Department of Geriatrics, The First Affiliated Hospital of Fujian Medical University, Fuzhou, People’s Republic of China; 2 Department of Cardiology, The First Affiliated Hospital of Gannan Medical University, Ganzhou, 341000 People’s Republic of China; 3 Key Laboratory of Prevention and Treatment of Cardiovascular and Cerebrovascular Diseases of Ministry of Education, Gannan Medical University, Ganzhou, 341000 People’s Republic of China; 4 Gannan Branch Center of National Geriatric Disease Clinical Medical Research Center, Gannan Medical University, Ganzhou, 341000 People’s Republic of China; 5 Fujian Hypertension Research Institute, The First Affiliated Hospital of Fujian Medical University, Fuzhou, People’s Republic of China; 6 Branch of National Clinical Research Center for Aging and Medicine, Fujian Province, The First Affiliated Hospital of Fujian Medical University, Fuzhou, China; 7 Fujian Provincial Clinical Research Center for Geriatric Hypertension Disease, The First Affiliated Hospital of Fujian Medical University, Fuzhou, China

**Keywords:** Pulmonary hypertension, Pulmonary artery smooth muscle cells proliferation, CREB, Protein phosphatases, Intracellular labile zinc

## Abstract

**Background:**

Transcription factor CREB is involved in the development of pulmonary hypertension (PH). However, little is known about the role and regulatory signaling of CREB in PH.

**Methods:**

A series of techniques, including bioinformatics methods, western blot, cell proliferation and luciferase reporter assay were used to perform a comprehensive analysis of the role and regulation of CREB in proliferation of pulmonary artery smooth muscle cells (PASMCs) in PH.

**Results:**

Using bioinformatic analysis of the differentially expressed genes (DEGs) identified in the development of monocrotaline (MCT)- and hypoxia-induced PH, we found the overrepresentation of CRE-containing DEGs. Western blot analysis revealed a sustained increase in total- and phosphorylated-CREB in PASMCs isolated from rats treated with MCT. Similarly, an enhanced and prolonged serum-induced CREB phosphorylation was observed in hypoxia-pretreated PASMCs. The sustained CREB phosphorylation in PASMCs may be associated with multiple protein kinases phosphorylated CREB. Additionally, hierarchical clustering analysis showed reduced expression of the majority of CREB phosphatases in PH, including regulatory subunits of PP2A, Ppp2r2c and Ppp2r3a. Cell proliferation analysis showed increased PASMCs proliferation in MCT-induced PH, an effect relied on CREB-mediated transcriptional activity. Further analysis revealed the raised intracellular labile zinc possibly from ZIP12 was associated with reduced phosphatases, increased CREB-mediated transcriptional activity and PASMCs proliferation.

**Conclusions:**

CREB pathway was overactivated in the development of PH and contributed to PASMCs proliferation, which was associated with multiple protein kinases and/or reduced CREB phosphatases and raised intracellular zinc. Thus, this study may provide a novel insight into the CREB pathway in the pathogenesis of PH.
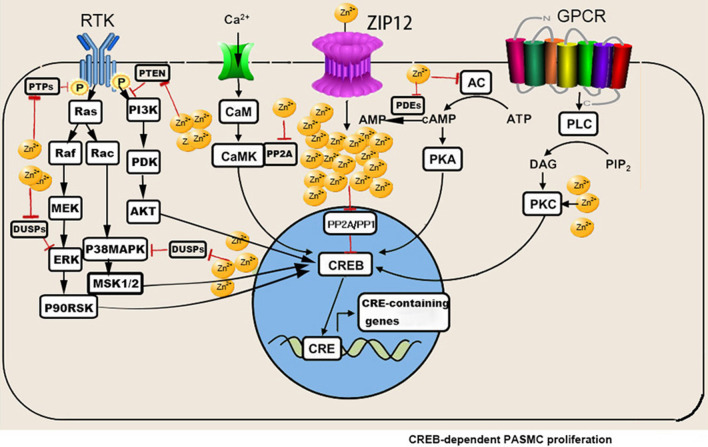

**Video abstract**

**Supplementary Information:**

The online version contains supplementary material available at 10.1186/s12964-021-00779-y.

## Plain English summary

Transcription factor CREB plays an important role in the development of pulmonary hypertension (PH). However, paradoxical roles have been reported in the pathogenesis of PH, and the regulatory mechanisms of CREB activation in pulmonary artery smooth muscle cells (PASMCs) proliferation remained unknown. In this study, we showed that CRE-containing genes were overrepresented among the differentially expressed genes in experimental PH, which resulted from the sustained activation of CREB pathway. The sustained activation of CREB pathway may be associated with the activation of multiple protein kinases that positively regulate CREB and down-regulation of numerous phosphatases involved in CREB dephosphorylation. Additionally, we found that the proliferation of PAMSCs was dependent on the CREB-mediated transcriptional activity in experimental PH. Moreover, the raised intracellular labile zinc possibly from ZIP12 may be associated with reduced protein phosphatases, increased CREB-mediated transcriptional activity and PASMCs proliferation. Collectively, we found CREB-mediated transcriptional activity in the proliferation of PASMCs in PH, which may be associated with multiple protein kinases and/or reduced phosphatases and elevated intracellular zinc. This study may reveal a critical role of zinc-mediated activation of CREB pathway in the proliferation of PASMCs, thus providing a more comprehensive understanding of CREB pathway in the pathogenesis of PH.

**Supplementary Information:**

The online version contains supplementary material available at 10.1186/s12964-021-00779-y.

## Background

Pulmonary hypertension (PH) is a cardiopulmonary vascular disease characterized by elevated pulmonary arterial pressure and pulmonary vascular remodeling, ultimately resulting in the right heart failure and premature death. The mortality remains high, despite therapeutic advances in management of patients with PH. Currently approved PH therapies attempt to restore the balance between vasodilator and vasoconstrictor mediators, but not reverse pulmonary vascular remodeling. Pulmonary vascular remodeling, mainly manifested as proliferation and migration of pulmonary artery smooth muscle cells (PASMCs) and extracellular matrix deposition, is a common feature of various types of PH. Hyperproliferative PASMCs are believed to be the critical pathogenic component of pulmonary vascular remolding [1, 2]. However, the underlying signaling pathways contributed to PASMCs proliferation and vascular remolding in PH were not well understood.

cAMP response element-binding protein (CREB) is a ubiquitously expressed transcription factor regulating the transcription of multiple genes in response to cAMP and cGMP [3]. The interaction of CREB with cAMP response element (CRE) is required for CREB-mediated gene transcription. CREB phosphorylation at Ser-133 promotes its binding at the CRE, recruitment of transcriptional coactivators CBP/p300 and consequently CREB-mediated transcription. CREB is known to regulate the expression of numerous genes important to the cardiovascular remodeling process [4]. For instance, CRE-dependent gene transcription played an important role in the survival and proliferation of vascular smooth muscle cells in vascular remodeling [5]. In addition, CREB mediated  tumor necrosis factor α (TNF-α)–induced vascular smooth muscle cell migration in vascular stenotic lesion [6].

CREB was reported to be selectively activated in the in vivo lung in response to hypoxia [7], and CREB expression and phosphorylation was increased in sugen5416/hypoxia-induced PH [8]. The elevated CREB phosphorylation was also identified in PASMCs derived from patients with idiopathic pulmonary arterial hypertension [9, 10]. Moreover, Nox1/Ref-1-mediated activation of CREB promotes gremlin1-driven endothelial cell proliferation and migration in sugen5416 + hypoxia-induced PH model [8]. However, the role of CREB in pulmonary vascular remodeling remains elusive, because either of the increased and reduced CREB expression has been proposed to mediate PASMCs proliferation in pulmonary vascular remodeling [9–13]. Additionally, the regulatory signaling of CREB activation in PASMCs proliferation has not been well characterized in PH.

Previously, we have showed the important role of CPT1, a CREB target gene, in regulating PASMC proliferation in PH [14]. In the present study, we applied a series of techniques, such as bioinformatics analysis, western blot and luciferase reporter assay to explore the expression and regulation of CREB in PH, aiming to provide a full understanding of the CREB pathway in proliferation of PASMCs. The results showed that the proliferation of PAMSCs was dependent on the CREB-mediated transcriptional activity and was associated with raised intracellular zinc and reduced protein phosphatases.

## Materials and methods

### Animal model

The procedures were approved by the Laboratory Animal Welfare and Ethics Committee of Fujian Medical University (Approval No. 2017–070, Fuzhou, China) and conducted in accordance with the ARRIVE guideline. Male Sprague–Dawley (SD) rats aged 8 weeks were purchased from Shanghai SLACCAS Laboratory Animal Co., Ltd (Certificate No. SCXK 2012–0002). The rats were raised with water and food ad libitum. The PH model in rats was induced by a single intraperitoneal injection of 40 mg/kg monocrotaline (MCT) (Sigma-Aldrich, CA, USA), control and MCT-treated rats were sacrificed at the end of week 1, 2, 3 and 4 after MCT treatment, as described in our previous study [15]. Mice in the hypoxic PH group were received a single subcutaneous injection of su5416 (20 mg/kg) and then exposed to a 10% oxygen hypoxic chamber for 8 h a day for 4 weeks, while control mice were exposed to indoor air. The mice were killed at the end of week 4, and lungs were immediately isolated for western blot analysis.

### Isolation, culture and treatment of PASMCs

The SD rats were anesthetized with 30 mg/kg sodium pentobarbital and killed by cervical dislocation. The isolation of PASMCs from the pulmonary arterioles was described in our previous study [14]. The identification of PASMCs was performed by staining of α-smooth muscle actin (α-SMA), a specific biomarker for vascular smooth muscle cells, using α-SMA antibody (Abcam, USA, 1:200). PASMCs were passaged after growth to 80%-90% confluence and starved with serum-free DMEM/F12 (Hyclone, Logan, UT, USA) for 24 h. Then, cells were pretreated with a variety of reagents, including protein kinase inhibitors (MedChemExpress LLC, USA), such as H89, SB203580, PD98059, LY294002, KN62, chelerythrine chloride and staurosporine. For the hypoxia treatment, the PASMCs were exposed to 2% O2 and 5% CO2 in sealed cell incubator (Thermo Scientific™ 8000, USA) for indicated times. The concentration of oxygen in the incubator was balanced by nitrogen and detected by an oxygen sensor inside the incubator. The PASMCs from generation 3 to 5 were used in the present experiments.

### Determination of cell proliferation

The cell proliferation was assessed by measurement of proliferating cell nuclear antigen (PCNA) expression by western blot and methyl thiazolyl tetrazolium bromide (MTT) assay. The MTT assay has been used in our previous study [14]. Briefly, PASMCs were seeded in the 96-well plates at a density of 1 × 10^4^ per well and cultured for 24 h. After treatment with the indicated reagents including TPEN (Sigma-Aldrich, USA) and FK506 (MedChemExpress LLC, USA), 20 μl MTT (5 mg/ml; Solarbio, Shanghai, China) was added to each well for 4 h of incubation. Then, the culture medium was discarded and 150 μl DMSO was added to each well in the plates. The plates were submitted to a microplate reader (Biotek, USA) for absorbance determination at 490 nm. The cell proliferation rate was determined by the absorbance ratio.

### RNA extraction and real-time PCR

Total RNA was isolated from 50 mg lung tissues and cultured PASMCs using Trizol reagent (Life Technology, USA) following manufacturer’s instructions [16]. The RNA concentration and purity were assessed at A260 nm and A280 nm wavelengths using NanoDrop™ instruments (Thermo Scientific, USA). Reverse transcription of total RNA to cDNA was performed according to the manufacturer’s protocol. The first-strand cDNA was used for real-time PCR. The forward and reverse primers for the amplification of each fragment were as follows: forward-5’-TGT CTG CTG ATC CAC GTG TC-3’, and reverse-5’-GAA ACG TCA TCC AGG GTC GT-3’ for ZIP12; and forward-5’-ACC ACA GTC CAT GCC ATC AC-3’, and reverse-5’-TCC ACC ACC CTG TTG CTG TA-3’ for GAPDH. Quantification of gene expression was performed using Light Cycler 96 (Roche, Switzerland). The relative quantification was performed by the comparative 2^−ΔΔCT^ method and expressed as fold changes.

### Total proteins extraction and western blot analysis

Lung tissues were homogenized in 1 × lysis buffer (2 × SDS, 5 × DTT, Cock tail 2 μl/ml, 100 × PMSF and H2O). PASMCs were cultured and treated in 6-well plates. When cells grew to 80–90% confluences, the medium was discarded. Then, PASMCs were washed three times with ice-cold phosphate buffered saline and lysed in 200 μl of 1 × lysis buffer. Tissue and cell lysates were incubated on ice for 30 min, subsequently centrifuged at 10,000 g for 15 min at 4 °C. After determination of protein concentration using the BCA protein assay kit (Beyotime, China), the supernatants were collected and mixed with equal volumes of 2 × SDS sample gel buffer. After boiling for 10 min, protein samples were separated by 10% SDS-PAGE and transferred to the polyvinylidene difluoride membranes. The membranes were washed 3 times with Tris Buffered Saline Tween (TBST), blocked at room temperature with 5% nonfat milk for 1 h, and incubated overnight at 4 °C with primary antibodies including anti-CREB (Cell Signaling Technology, 1:1000), anti-phosphorylated-CREB (Cell Signaling Technology, 1:1000), anti-PCNA (Abcam, 1:1000), anti-β-actin (Santa Cruz, 1:500) and anti-ZIP12 (HuaBio, Co., Ltd. China, 1:1000). Anti-ZIP12 antibody was produced following the methods described in the previous study [17], by HuaAn Biotechnology, Co., Ltd., Hangzhou, China. The membranes were then washed 3 times with TBST and incubated with horseradish peroxidase-conjugated secondary antibodies (Santa Cruz, 1:2000) for 1 h at 37.5˚C. After being washed and revealed with the ECL detection kit (Beyotime, China), the bands were visualized by exposure to X-ray film, scanned and quantified by Image J software.

### Immunofluorescent staining

Immunofluorescence staining was described previously [14]. The deparaffinized and dehydrated sections (5 μm) from lung tissues or glass coverslips with cells were fixed with 4% formaldehyde in phosphate buffer saline (PBS) for 10 min at room temperature. The samples were rinsed with PBS, treated with 0.2% Triton X-100 on ice for 10 min for permeabilization and then blocked with 5% non-fat milk for 30 min at room temperature. The slides were incubated with anti-phosphorylated-CREB antibody (Cell Signaling Technology, 1:200) and anti-alpha smooth muscle actin antibody (α-SMA, Abcam, 1:400) in blocking solution overnight. Next, the samples were washed three times with PBS and incubated with goat anti-rabbit IgG FITC (1:100, ZSGB-Bio, China) and goat anti-mouse IgG TRITC (1:100, ZSGB-Bio, China) in PBS for 2 h at room temperature. The slides were washed three times with PBS before being incubated with 4’,6-diamidino-2-phenylindole (DAPI) (1 μg/mL; Santa Cruz Biotechnology, USA) in PBS for 5 min. The specimens were mounted in 90% glycerol, sealed with nail polishoil, and observed under inverted phase-contrast immunofluorescent microscope (Ts2R/FL, Nikon, Japan).

### Construction of pGL4.20-TH reporter by ligation-independent cloning

Tyrosine hydroxylase (TH) transcription depends primarily on CRE activity, regardless of the types of inducing stimulus [18]. The TH promoter (-4490 ~  + 10) was linked to a luciferase reporter, pGL4.20 (Vazyme Biotech Co.,Ltd, China), to construct pGL4.20-TH (rat) by Anti-hela Biological Technology, Xiamen Co. Ltd, China. The construction of pGL4.20-TH reporter was briefly described as follows: 1) TH promoter (− 4490– + 10) was amplified from rat genome by using the primers: forward, 5’-AAC TGG CCG GTA CCg aag gaa aga tct cca ggg c-3’, and reverse, 5’-TCT TGA TAT CCT CGA Gtt tcc act ggg ttt taa tat g-3’. 2)The pGL4.20 vector was digested using KpnI and XhoI at 37˚C for 2 h. 3) After purification and retrieval of DNA fragments, the amplified fragments and digested vector were mixed with ExoIII, placed on the ice for 1 h, and then added 1μL 0.5 M EDTA and melted at 70˚C for 5 min to stop the reaction. 4) After *E.coli *DH5α transformation and culture, a single clone was selected and grown in LB medium for plasmid preparation. 5) The sequences of pGL4.20-TH constructs were verified by NextGen Sequencing (Sangon, China). The eligible pGL4.20-TH plasmid and pGL4.20 empty vector were chosen to amplify and purify for further use.

### Transfection and CRE-luciferase assay

PASMCs were seeded in 24-well plates. After different treatments, cells at about 60% confluence were transfected with 1.3 μg of PGL4.2 TH-promoter plasmid together with 0.2 μg of PGL4.74-Rluc plasmid using Lipofectamine 2000 (Life Technologies, USA). Then PASMCs were lysed and the cell lysates were used for detection of CRE-luciferase activity. The Luciferase activity was determined by using Dual-Luciferase Reporter Gene Assay (Vazyme Biotech Co.,Ltd, China), according to the manufacturer’s instructions.

### The overexpression of cAMP response element modulator (CREM)

Small isoforms of CREM, in particularly ICER, inhibit CREB-mediated transcriptional activity [19]. Premade adenovirus vector expressing ICER (Ad-CREM, gene sequence number NM_182769) was purchased from Vigene biosciences, USA. PASMCs were seeded and cultured in 6-well plates. When PASMCs reached 80%–90% confluence, PASMCs were infected with 0.1 μl green fluorescent protein-empty adenovirus vector (Ad-GFP, Vigene biosciences, USA) or 10 μl Ad-CREM at a multiplicity of infection of 100, in the present of 4 μl virus co-infection reagent ADV-HR (Vigene biosciences, USA).

### Measurements of intracellular zinc levels

Intracellular labile zinc levels were measured by using the cell-permeable zinc specific dye FluoZin™-3, AM (Life Technologies, USA). PASMCs were seeded on coverslips in 6-well plates. When the cells were near to 70% confluent, the medium was removed. PASMCs were washed twice by the balanced salt solution and incubated with the Fluozin-3 AM for 30 min at 37 °C. Then, the cells were washed twice with balanced salt solution to remove excess probe. Fluorescence emission intensity was detected by fluorescence microscope (Olympus, Inc., Tokyo,Japan) at the wavelength of 515 nm after excitation at 495 nm.

### RNA sequencing and bioinformatics analysis

cDNA library preparation and RNA sequencing were described in our previous study [15]. The raw RNA sequencing data that support the findings were deposited in the gene expression omnibus repository with an accession number GSE149713. Dataset of phosphatase profiling in hypoxia and hypoxia + su5416-induced PH was downloaded and extracted from gene expression omnibus repository with an accession number GSE8078 (https://www.ncbi.nlm.nih.gov/geo/query/acc.cgi?acc=GSE8078). GSE8078 dataset was comprised of 3 sample groups, including normoxia/vehicle (n = 4), hypoxia/vehicle (n = 4), hypoxia/su5416 (n = 3) [20]. Heatmap creation and hierarchical clustering of DEGs were conducted by Morpheus (https://software.broadinstitute.org/morpheus/) and ClustVis (https://biit.cs.ut.ee/clustvis/). The identification of CRE-containing genes was performed by use of a searchable database called CREB Target Gene Database (http://natural.salk.edu/CREB) [21].

### Statistical analysis

Data were showed as mean ± S.E.M. GraphPad Prism 8 was used for statistical analysis. Comparison of two groups was performed by using unpaired *t* test. For multiple comparisons, the data were analyzed by using one-way ANOVA, and followed by the Turkey test within groups. A *p* value of < 0.05 was considered as statistical significance.

## Results

### Involvement of CREB-mediated transcription in the development of PH

**Fig. 1:**
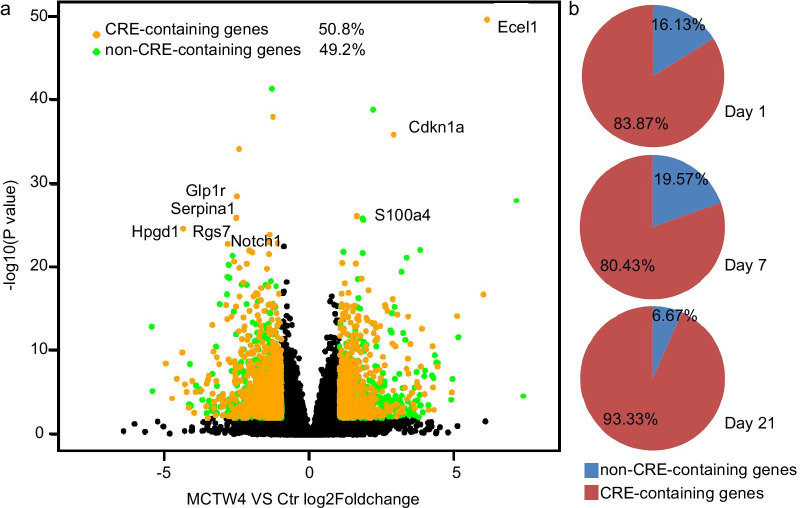
CRE-containing genes among DEGs induced by MCT and hypoxia. **a** Volcano plot showing overrepresention of CRE-containing DEGs in MCT-induced PH. **b** The percentage of CRE-containing DEGs in hypoxia-induced PH. The DEGs induced by hypoxia were derived from Kwap iszewska et al. Respir Res, 2005. 6: p.109. CRE-containing genes were identified by searching CREB target gene database (http://natural.salk.edu/CREB)

Our previous transcriptomic analysis of MCT-induced PH showed differential expression of 280, 1342, 908 and 3155 genes at the end of week 1, 2, 3 and 4 after MCT treatment [15]. Because the CRE site in a gene promoter is a prerequisite for the CREB-mediated transcription, we examined the CRE sites in the differentially expressed genes (DEGs) at week 4. Analysis of DEGs through searching CREB Target Gene Database showed that 50.8% DEGs have one or more CRE sites in their promoters, the corresponding volcano plot showed that CRE-containing DEGs were highly overrepresented among DEGs at week 4 (Fig. 1a). To investigate the relevance of MCT-induced PH model to hypoxia-induced PH model, we examined the DEGs in the development of hypoxia-induced PH. A total of 29, 38 and 42 DEGs were identified in mice subjected to 1, 7 and 21 days of hypoxia, respectively [22]. Further analysis of the DEGs showed that 83.87%, 80.43% and 93.33% of DEGs have the CRE sites in their promoters at the corresponding time point, respectively (Fig. 1b). As a result, these results suggested the involvement of CREB-mediated transcription in the development of PH.

### Verification of the activated CREB pathway in PH

**Fig. 2:**
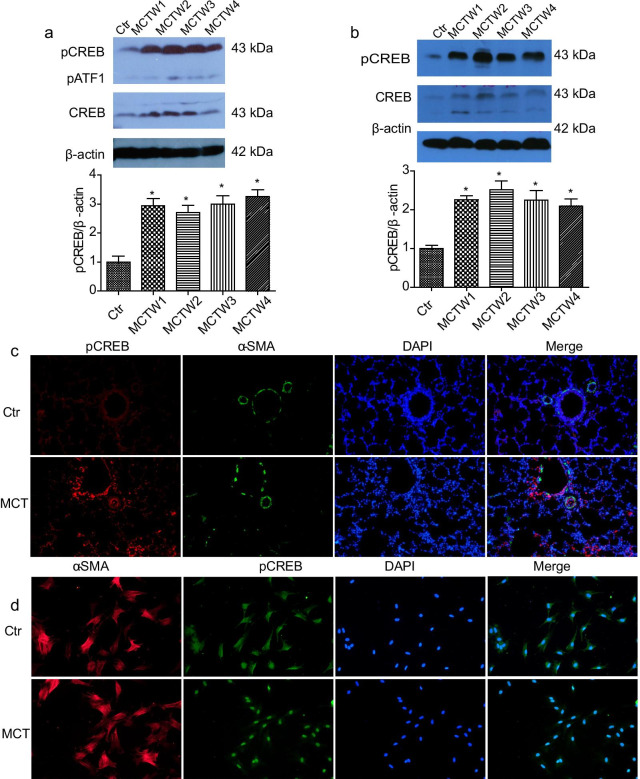
The effect of MCT on the expression and phosphorylation of CREB. **a** The elevated CREB phosphorylation in lung tissues. The lung tissues were isolated from control and rats treated with MCT for 1, 2, 3 and 4 weeks. n = 7. **b** The CREB phosphorylation and expression in PASMCs derived from control and rats treated with MCT for 1, 2, 3 and 4 weeks. n = 7. **c** Immunofluorescence staining showed the increased phosphorylation of CREB (red staining) in the distal pulmonary arteries. n = 5. **d** The increased entry of phosphorylated-CREB (green staining) in PH-PASMCs, revealed by immunofluorescence staining. n = 3. *p < 0.05 versu Ctr

CREB-mediated transcription requires phosphorylation at CREB serine-133, thus, we determined whether CREB serine-133 phosphorylation was increased in MCT-induced PH. Total- and phosphorylated-CREB were determined by western blot. A sustained increase in phosphorylated- and total-CREB were observed in rat lungs and corresponding PASMCs (Fig. 2a, b and Additional file 1: Figure S1a, b), indicating a role of CREB in the development of MCT-induced PH. To assess the phosphorylated-CREB level in the distal pulmonary arteries, immunofluorescence staining was performed in the lung tissues and PASMCs. As showed in the Fig. 2c, CREB phosphorylation was increased in the distal pulmonary arteries by immunofluorescence. Moreover, immunofluorescence staining also demonstrated an increased entry of phosphorylated-CREB in the nuclear of PASMCs isolated from rats with PH (Fig. 2d). Collectively, these results indicated that CREB pathway was activated in the development of MCT-induced PH.

Hypoxia is a stimulating factor for induction of pulmonary vasoconstriction and pulmonary vascular remodeling. To determine whether CREB was activated in hypoxia + su5416-induced PH model, we isolated the lung tissues and examined the phosphorylated CREB. Consistently, western blot analysis showed that CREB phosphorylation was increased in hypoxia + su5416-induced PH (Additional file 1: Figure S1c). To assess the role of hypoxia in CREB phosphorylation in PASMCs, the PASMCs isolated from control rats were exposed to hypoxia (2% O_2_) for various times. It was showed that CREB phosphorylation and expression were not directly affected by hypoxia (Additional file 2: Figure S2a). 10% serum (FBS) treatment induced CREB phosphorylation in a time-dependent manner with a peak phosphorylation at 15 min, and soon afterwards declined near to baseline levels at 1 h (Additional file 2: Figure S2b). To determine whether the lack of CREB phosphorylation in response to hypoxia was due to the absence of serum, PASMCs were grew in 10% serum and then exposed to hypoxia. Similarly, hypoxia treatment did not directly increase the CREB phosphorylation in 10% serum cultured PASMCs (Additional file 2: Figure S2c). To determine whether hypoxia indirectly affected the CREB phosphorylation, the PASMCs were cultured in serum-free medium and pretreated with 2% O_2_ for 24 h, and then treated with 10% serum for the indicated times. Interestingly, pretreatment of PASMCs with hypoxia for 24 h exhibited enhanced and prolonged serum-induced CREB phosphorylation (Additional file 3: Figure S3a, b). Taken together, these results suggested that the effect of hypoxia on CREB phosphorylation in PASMCs was not direct but indirect.

### Identification of potential protein kinases involved in CREB phosphorylation

**Fig. 3:**
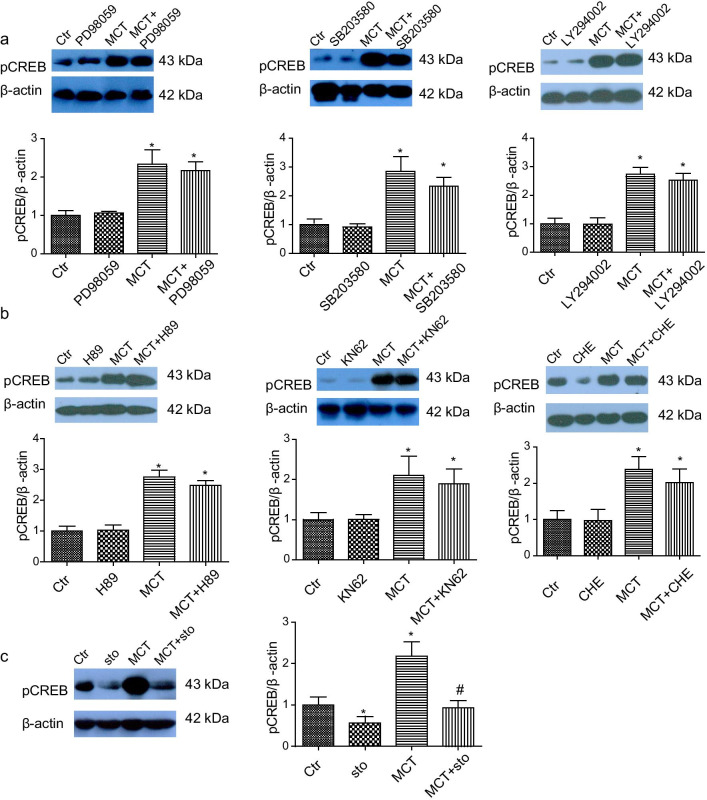
Identification of potential protein kinases involved in CREB phosphorylation. **a** The effect of inhibition of ERK1/2, P38MAPK and PI3K on CREB phosphorylation in PH-PASMCs. The PH-PASMCs were starved with serum-free DMEM/F12 for 24 h, and then the cells were pretreated with ERK1/2 inhibitor PD98059 (20 μM), P38MAPK inhibitor SB203580 (20 μM) and PI3K inhibitor LY294002 (50 μM) for 24 h, respectively. n = 4, 7 and 5. **b** The effects of inhibition of PKA, CAMKII and PKC on CREB phosphorylation in PH-PASMCs. The PH-PASMCs were starved with serum-free DMEM/F12 for 24 h, and then the cells were pretreated with PKA inhibitor H89 (10 μM), CaMK inhibitor KN62 (10 μM) and PKC inhibitor chelerythrine chloride (CHE, 10 µM) for 24 h, respectively. n = 5, 7 and 5. **c** The effect of staurosporine (Sto, 10 nM) on CREB phosphorylation in PH-PASMCs. The PH-PASMCs were starved with serum-free DMEM/F12 for 24 h, and then were pretreated with Sto for 1 h. n = 4. Ctr, control; MCT, MCT treatment for 4 weeks. *p < 0.05 vs. Ctr, #*p* < 0.05 vs. MCT group

A variety of protein kinases including PKA, P38MAPK, ERK1/2, Akt, CaMK and PKC have been reported to phosphorylate CREB [23]. To identify the upstream kinases involved in CREB phosphorylation in MCT-induced PH, we pretreated PASMCs isolated from rats with PH (PH-PASMCs) with various protein kinase inhibitors, including PKA inhibitor H89, P38MAPK inhibitor SB203580, ERK1/2 inhibitor PD98059, PI3K inhibitor LY294002, CaMK inhibitor KN62 and PKC inhibitor chelerythrine chloride (CHE). It was showed that the sustained CREB phosphorylation in PH-PASMCs was not markedly attenuated by these protein kinase inhibitors (Fig. 3a, b). Similarly, the enhanced and prolonged CREB phosphorylation in hypoxia-pretreated PASMCs were not significantly attenuated by these kinase inhibitors (Additional file 4: Figure S4a, b). The less effect of protein kinase inhibitors alone on CREB phosphorylation led to the possibility that sustained CREB phosphorylation was caused by multiple protein kinases. Then, we pretreated the PASMCs with staurosporine, a multiple protein kinase inhibitor [24]. As shown in Fig. 3c and Additional file 4: Figure S4c, the CREB phosphorylation was almost completely abolished by staurosporine in both PH-PASMCs and hypoxia-pretreated PASMCs.

### Identification of reduced phosphatases associated with CREB dephosphorylation

**Fig. 4:**
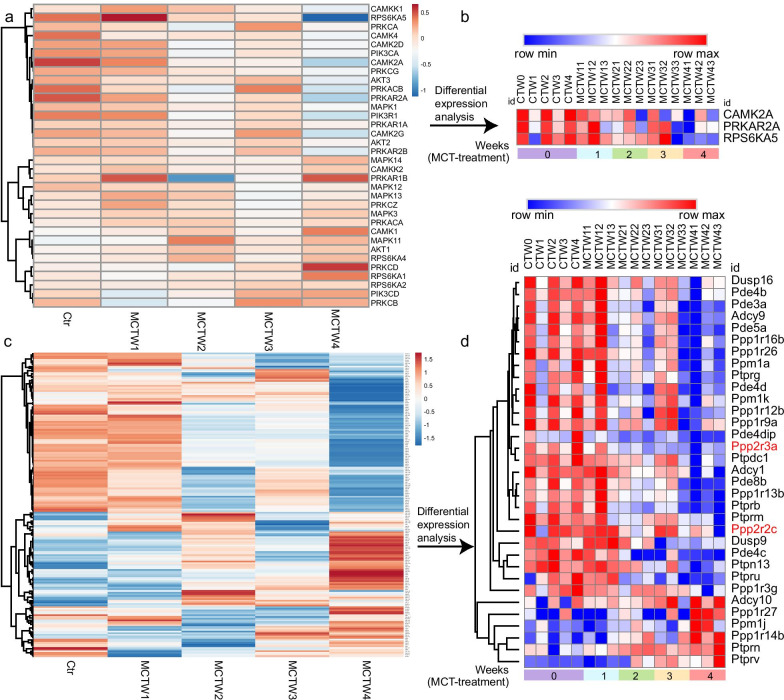
Profiling of protein kinases and phosphatases associated with CREB phosphorylation in MCT-induced PH. **a**, **c** Hierarchical clustering of the protein kinases and phosphatases related to CREB phosphorylation by using ClustVis. Rows in the heatmap represent averaged gene expression of replicates and columns represent time point. n = 3 MCT-treated rats at each time point and n = 5 control. **b**, **d** Heatmap of differentially expressed protein kinases and phosphatases induced by MCT. Rows in the heatmap represent gene expression levels and columns represent each sample. The DEGs between control and MCTW4 were identified by using a threshold of fold change ≥ 2 and *p* ≤ 0.05. Ctr, control; MCTW1, MCTW2, MCTW3 and MCTW4 represent MCT treatment for 1, 2, 3 and 4 weeks, respectively

To further evaluate the protein kinases in CREB phosphorylation, we determine the expression of protein kinases in MCT-induced PH. Hierarchical clustering of the protein kinases identified in our RNA-seq dataset using ClustVis showed that the majority of protein kinases seemed to be downregulated (Fig. 4a). Differential expression analysis showed that only the CaMK subunit CAMK2A, PKA subunit PRKAR2A, and MSK1/2 subunit RPS6KA5 were differentially expressed, however, they were downregulated in the development of PH (Fig. 4b), thus indicating less important role of protein kinases in regulating sustained CREB phosphorylation. Of note, CREB phosphorylation was regulated not only by protein kinases, but also by phosphatases that antagonize the CREB phosphorylation. To determine whether phosphatases have a role in regulating CREB phosphorylation, the potential enzymes related to CREB dephosphorylation were extracted from our RNA-seq dataset, including PP2A, PP1, PDEs, protein tyrosine phosphatases (PTPs) and dual specificity phosphatases (DUSPs). Hierarchical clustering of CREB phosphatases using ClustVis showed the majority of phosphatases have a trend to be reduced, with the maximal numbers at 4 weeks of MCT treatment (Fig. 4c). Differential expression analysis using DESeq2 package identified several phosphatases that were differently expressed in comparison of MCT-treatment 4 weeks with control, including 19 downregulated phosphatases and 6 upregulated phosphatases. The downregulated phosphatases included regulatory subunits of PP2A and PP1, PTPs, as well as DUSPs (Additional file 5: Table S1). The adenylyl cyclases that positively regulated cAMP production were also downregulated.

Hierarchical clustering of the differentially expressed phosphatases using Morpheus revealed the sustained reduction in the expression of PP2A regulatory subunits Ppp2r2c and Ppp2r3a, especially for Ppp2r3a whose expression pattern was in accordance with the sustained CREB phosphorylation in the development of MCT-induced PH (Fig. 4d). In contrast to PP2A, the expression of PP1 varied with subunits: Ppp1r27 and Ppp1r14b expression were upregulated, whereas the other PP1 regulatory subunits, including Ppp1r12b, Ppp1r16b, Ppp1r13b, Ppp1r26, Ppp1r3g and Ppp1r9a were downregulated (Additional file 5: Table S1 and Fig. 4d). The increased CREB phosphorylation has been observed in hypoxia + su5416-induced PH model from the present study. Similar to MCT-induced PH, hierarchical clustering of CREB phosphatases in hypoxia- and hypoxia + su5416-induced PH revealed decreased expression of phosphatases, including the subunits of PP2A (Ppp2r3a and Ppp2r2c), PP1 and PTPs (Additional file 6: Figure S5). Collectively, these results indicated that the sustained CREB phosphorylation in PH may be related to the reduced phosphatases that dephosphorylate CREB.

### The effect of CREB-mediated transcriptional activity on PASMCs proliferation

**Fig. 5:**
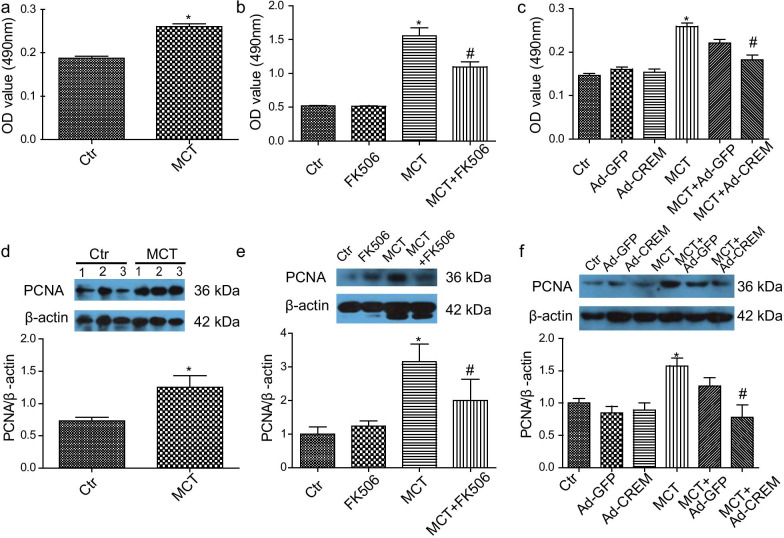
The effect of CREB-mediated transcriptional activity on PASMCs proliferation. **a**, **d** The elevated cell proliferation in PH-PASMCs. The PASMCs were isolated from control and MCT-treated rats. n = 6, 4. **b**, **e** The inhibitory effect of FK506 on cell proliferation. Cells were starved with serum-free DMEM/F12 for 24 h, and then treated with FK506 (1 nM) for 24 h. n = 6, 5. **c**, **f** The inhibitory effect of CREM on cell proliferation. Cells were starved with serum-free DMEM/F12 for 24 h and transfected with Ad-CREM and Ad-GFP for 24 h. n = 6, 5. Cell proliferation was determined by MTT assay (a-c) and measurement of PCNA expression (**d**–**f**). Ad-CREM, ICER-expressing adenovirus vector; Ad-GFP, green fluorescent protein-empty adenovirus vector. Ctr, control; MCT, MCT treatment for 4 weeks. *p < 0.05 vs. Ctr, ^#^*p* < 0.05 vs MCT

Pulmonary vascular remodeling in PH is characterized by hyperproliferation and migration of PASMCs. Because CREB phosphorylation alone is not a reliable predictor of target gene activation [21, 25], in the present study we sought to assess the effect of CREB-mediated transcriptional activity on PASMCs proliferation. As shown in Fig. 5a, MTT assay showed the increased proliferation of PH-PASMCs, and the cell proliferation was attenuated by FK506 (Fig. 5b). To further determine cell proliferation in PH-PASMCs, cell proliferation was assessed by measurement of PCNA, a marker of cell proliferation. Similarly, it was showed that PCNA expression was significantly increased in PH-PASMCs, a response that was reversed by FK506 (Fig. 5d, e). Because FK506 was the inhibitor of CREB-mediated gene transcription [26–29], the inhibition of cell proliferation by FK506 suggested a role of CREB-mediated transcriptional activity on PASMCs proliferation. CREM acts as the negative regulator of CREB-mediated transcriptional activity via competitive CRE binding site in a gene promoter [30]. In further support of the effect of CREB-mediated transcriptional activity on PASMCs proliferation, we transfected PASMCs with a recombinant adenovirus vector expressing ICER (Ad-CREM) and an empty adenoviral vector (Ad-GFP). Consistently, adenoviral mediated overexpression of ICER inhibited PH-PASMCs proliferation, as determined by both detection of MTT and PCNA methods (Fig. 5c, f). As a result, these results further indicated that PASMCs proliferation in MCT-induced PH was relied on CREB-mediated transcriptional activity.

### Role of zinc in regulation of CREB-mediated transcription and cell proliferation

**Fig. 6:**
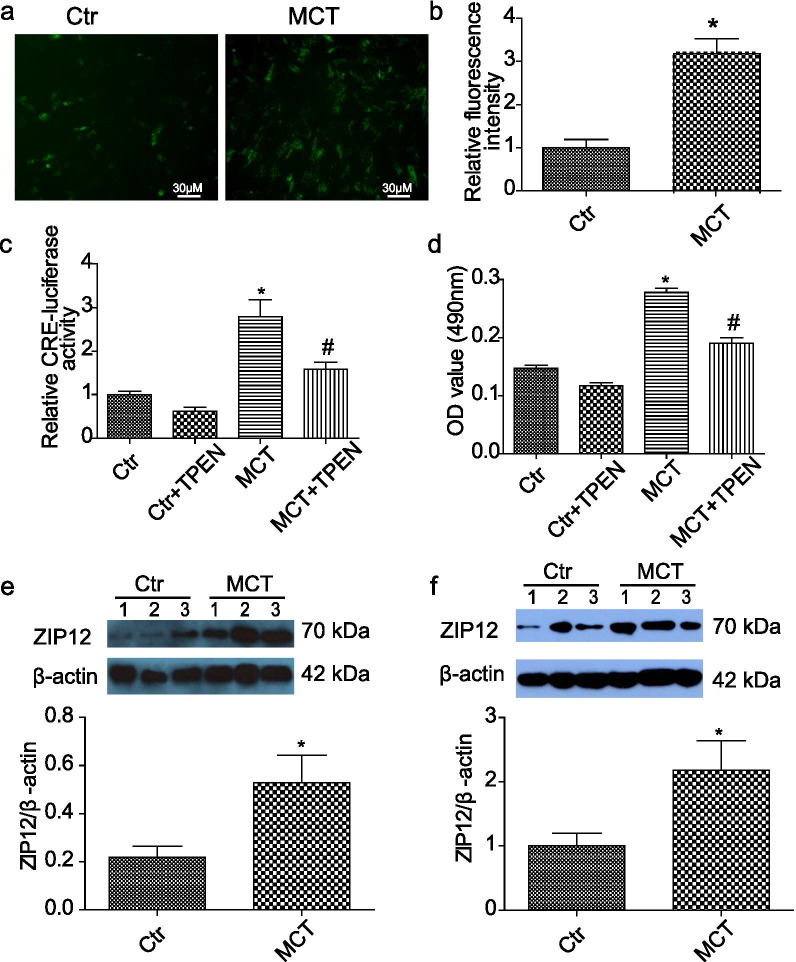
Role of zinc in regulation of CREB-mediated transcription and cell proliferation. **a** Representative zinc probe fluorescence images in PASMCs isolated from control and MCT-treated rats. The intracellular labile zinc levels in PASMCs were stained by fluozin-3. **b** Summarizing the relative intensities of fluorescence in PASMCs. n = 4. **c** The effect of zinc on CRE-luciferase reporter activity in PH-PASMCs. PASMCs were infected with pGL4.20-TH reporter, an adenoviral vector expressing a CRE-luciferase. n = 4. **d** The effect of zinc on PASMCs proliferation determined by MTT. Serum starved cells were pretreated with TPEN (10 μM) for 24 h. n = 6. TPEN, N,N,N′,N′-Tetrakis (2-pyridylmethyl) ethylenediamine; Ctr, control; MCT, MCT treatment for 4 weeks. *p < 0.05 vs. Ctr, ^#^*p* < 0.05 vs. MCT. **e** Elevated expression of ZIP12 protein in rat lung homogenate. n = 8. **f** Elevated expression of ZIP12 protein in isolated PH-PASMCs. n = 4. Ctr, control; MCT, MCT treatment for 4 weeks. *p < 0.05 vs. Ctr

The finding that numerous downregulated protein phosphatases in MCT-induced PH were reminiscent of the labile zinc which was well known to inhibit phosphatases including PTPs [31–33], PTEN [34], DUSPs (MKPs) [35], PP2A [36, 37], etc. As expected, measurement of zinc using fluozin-3 showed that intracellular labile zinc was elevated in PH-PASMCs (Fig. 6a, b). In addition, intracellular zinc was reported to regulate CREB-mediated transcriptional activity [38]. To determine whether intracellular zinc has a role in regulating CREB-mediated transcriptional activity in PH-PASMCs, we constructed the pGL4.20-TH luciferase reporter that has the CRE-luciferase activity (Additional file 6: Figure S6a). In line with the elevated CREB phosphorylation, the CREB-mediated transcriptional activity, represented by the pGL4.20-TH luciferase reporter activity, was increased in PH-PASMCs, while zinc chelation using TPEN resulted in the reduced pGL4.20-TH luciferase activity (Fig. 6c), thus, indicating the role of intracellular zinc in regulating CREB-mediated transcriptional activity in PH-PASMCs. Moreover, we found that chelation of intracellular zinc by TPEN also led to abolish PH-PASMCs proliferation (Fig. 6d), thus suggesting a role of zinc in regulating PASMCs proliferation. Taken together, the above results, in combination of PASMCs proliferation relied on CREB-mediated transcriptional activity, indicated a role for intracellular zinc in regulating PASMCs proliferation via CREB-mediated transcriptional activity. Zinc transporter ZIP12 was reported to raise the intracellular zinc and mediate cell proliferation in hypoxia-treated PAMSCs [17]. To determine whether ZIP12 played a role in MCT-induced PH, we examined the ZIP12 expression in both mRNA and protein levels. Quantitative PCR analysis showed a marked increase in ZIP12 mRNA expression in both lung tissues and PH-PASMCs (Additional file 7: Figure S6b, c). Western blot analysis confirmed the significant upregulation of ZIP12 protein levels in lung tissues and PH-PASMCs (Fig. 6e, f). Furthermore, we found that ZIP12 was markedly upregulated in hypoxia + su5416-induced PH (Additional file 7: Figure S6d).

## Discussion

In the present study, we found that PAMSCs proliferation was dependent on the CREB-mediated transcriptional activity in MCT-induced PH. CRE-containing genes were overrepresented among the DEGs, which resulted from the sustained activation of CREB pathway. The sustained CREB phosphorylation was associated with the activation of multiple protein kinases and/or down-regulation of phosphatases. Additionally, the raised intracellular labile zinc possibly from ZIP12 may play a role in mediating CREB transcriptional activity and PASMCs proliferation.

We found the elevated proliferation of PASMCs in MCT-induced PH. Because CREB phosphorylation alone was not a reliable predictor of target gene transcription [21, 25], we did not seek to assess CREB phosphorylation on PASMCs proliferation, instead, we evaluated CREB-mediated transcriptional activity on PASMCs proliferation through two methods: inhibition of CREB-mediated transcriptional activity by FK506 and overexpression of CREB negative regulator CREM. Our results demonstrated a role of CRE-mediated transcriptional activity in PASMCs proliferation.

CREB was originally identified, due to its response to cAMP signaling, while prostacyclin and its analogues, the widely used drug for treatment of patients with PH, increases intracellular cAMP formation via adenylyl cyclase stimulation. The paradoxical role of CREB in pulmonary vascular remolding and PH was reported in PH. The CREB knockout in animals may provide better understanding of the role of CREB in the PH pathogenesis. CREB have three functional isoforms alpha, beta and delta, produced by alternative splicing. Due to perinatal lethality in mice deleted all isoforms of CREB [39], the mice with complete deletion of CREB may not be used to investigate the role of CREB in PH. However, the mice with deletion of alpha and delta isoforms of CREB are viable, owing to an overexpression of the CREB beta isoform [40]. The mice with deletion of alpha and delta isoforms of CREB have already been used to investigate the role of CREB in PH [41]. Hypoxia selectively activates the CREB family of transcription factors in the in vivo lung, contributing to pulmonary vascular remodeling and PH [7]. Unexpectedly, the same group revealed that deletion of alpha and delta isoforms of CREB in mice contributed to elevated pulmonary vascular resistance both in normoxia and following exposure to hypoxic conditions for three weeks [41]. They concluded that alpha and delta isoforms of CREB regulate homeostatic gene expression in the lung and that normal activity of these isoforms is essential to maintain low pulmonary vascular resistance and to maintain the normal alveolar structure [41]. In fact, this paradoxical role of CREB was also appeared in the other conditions [4, 42]. Both VEGF and PAI-1 have a CRE site in their gene promoters, beraprost, a prostacyclin analogue used for PH treatment, increases VEGF and decreases PAI-1 expression through PKA/CREB pathway in vascular smooth muscle cells [43]. Similarly, we were able to identify the CRE motif within the promoters of 50.8% DEGs, including the downregulated and upregulated DEGs. The exact mechanisms underlying these divergent results remained unclear. However, the divergent results may be explained by the feature of bidirectional promoters. Previous analysis of genome-wide CREB binding events showed occurrence of CREB binding sites in bidirectional promoters, and CREB regulated bidirectional transcription [44]. The subsequent study revealed a strong preference for CREB binding at these bidirectional promoters, when CREB was bound at the bidirectional promoters, promoter activity in one direction was upregulated, whereas promoter activity in the other direction was repressed [45].

CREB ser-133 phosphorylation allows recruitment and binding of transcriptional coactivators CBP/p300 and subsequently, CREB target genes transcription. In this study, we found a sustained increase in CREB phosphorylation in the lungs of MCT-treated rats. CREB phosphorylation is regulated by multiple protein kinases, including PKA, ERK1/2, P38MAPK, Akt, CaMK and PKC. Although the concentration of the protein kinase inhibitors used in this study were in accordance to with the instructions provided by the manufacturers and proved to be effective, pretreatment of PASMCs with various protein kinase inhibitors alone failed to significantly inhibit sustained phosphorylation of CREB, thus coincided with previous study showing failure to determine the exact upstream protein kinases in hypoxia-treated PC12 cells [46]. In the present study, the sustained CREB phosphorylation was almost completely abolished by staurosporine, a multiple protein kinase inhibitor, indicating that multiple protein kinases may be involved in CREB phosphorylation and/or the compensatory mechanisms existed in protein kinases that phosphorylated CREB. However, the identification of downregulated multiple protein kinases indicated limited effect of protein kinases on sustained CREB phosphorylation.

Most studies on CREB pathway focused on the protein kinases. However, the CREB phosphorylation was also regulated by protein phosphatases and few studies have evaluated the CREB phosphatases in the development of PH. In this study, a number of phosphatases, including the subunits of PP2A, PP1, PTPs and DUSPs were found to be reduced in MCT-induced PH. PP2A was the phosphatases responsible for directly dephosphorylating in phosphorylated CREB [47]. PP2A is consisted of a structural A subunit, a catalytic C subunit and a regulatory B subunit. The regulatory B subunit dictates subcellular localization and substrate specificity, and 20 different regulatory subunits have been identified. We found that 2 regulatory B subunits of PP2A were reduced, including Ppp2r2c and Ppp2r3a. The Ppp2r2c and Ppp2r3a determine the substrate selectivity and catalytic activity[48], The Ppp2r3a expression was persistently downregulated with a pattern explained the sustained CREB phosphorylation. Therefore, it is likely that the reduced expression of PP2A regulatory subunits, in particular Ppp2r3a, may account for the impaired PP2A activity observed by the previous study in PASMCs from patients with pulmonary arterial hypertension [49]. PP1 phosphatase was also known as the CREB phosphatases [50]. Multiple regulatory subunits of PP1 were identified as differential expression in this study, including upregulated and downregulated subunits. The role of PP1 in the dephosphorylation of CREB remained unknown, due to simultaneous identification of upregulated and downregulated inhibitory subunits of PP1 in MCT-induced PH. Additionally, a variety of PTPs that negatively regulated RTKs signaling were downregulated. Interestingly, hypoxia decreased expression of numerous PTPs, including SHP-2, DEP-1, and PTP-1B in PASMCs, resulting in the reduced PTPs activity in hypoxia-induced PH [51]. The typical CREB phosphorylation elicited by stimulus such as TNFα is a rapid response that then declines [6, 46]. In the present study, it was showed that hypoxia enhanced and prolonged serum-induced CREB phosphorylation. It was possible that the prolonged and enhanced serum-induced CREB phosphorylation induced by hypoxia may be due to the reduced phosphatases that negatively regulate CREB phosphorylation.

**Fig. 7:**
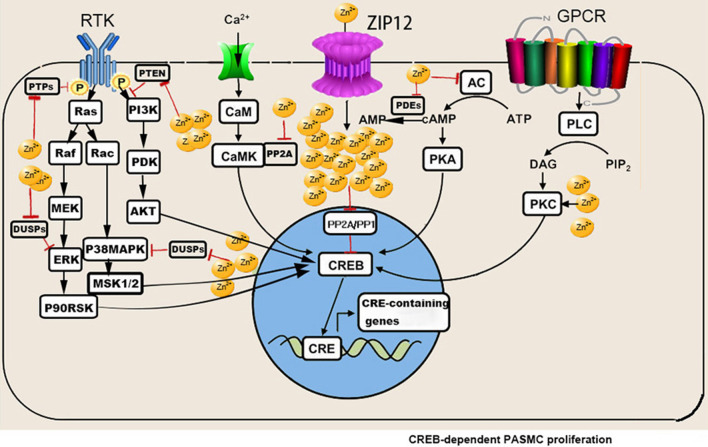
A proposed model for the role and regulation of CREB in the proliferation of pulmonary artery smooth muscle cells in pulmonary hypertension

The labile zinc was reported to regulate CREB activation [38, 52] and inhibit protein phosphatases [32, 35, 36]. The reduced expression of multiple protein phosphatases were identified and may be associated with elevated intracellular zinc in MCT-induced PH. As an intracellular signaling mediator, zinc is involved in a variety of biological processes. Interestingly, a role for labile zinc in regulating hypoxic pulmonary vasoconstriction has been reported [53, 54]. The ZIP12 is localized to the plasma membrane and capable of mediating the influx of extracellular zinc into the cytosol. Recently, ZIP12 was reported to promote cell proliferation in hypoxia-treated PASMCs through elevation of the intracellular labile zinc [17]. In addition, intracellular zinc derived from ZIP12 has been reported to regulate neurulation and neurite extension through CREB activation [38]. In line with a recent study [55], the upregulation of ZIP12 was observed in MCT-induced PH. Furthermore, we confirmed that ZIP12 was upregulated in hypoxia + su5416-induced PH. From the present study, it could be speculated that intracellular zinc mediated by ZIP12 may contribute to PASMCs proliferation through regulation of CREB-mediated transcriptional activity (Fig. 7). However, further research is still needed to investigate the role of ZIP12-mediated zinc in regulating phosphatase activity and CREB transcriptional activity in PH.

## Conclusions

In summary, we found CREB-mediated transcriptional activity in the proliferation of PASMCs in PH, which may be associated with multiple protein kinases and/or reduced phosphatases and elevated intracellular zinc. This study may indicate a critical role of zinc-mediated activation of CREB pathway in the proliferation of PASMCs, thus providing a more comprehensive understanding of CREB pathway in the pathogenesis of PH.

## Supplementary Information

**Additional file 1: Figure S1**. Total and phosphorylated CREB expression in lung and PASMCs. **a** Lung tissues were isolated from control and rats induced by MCT for 1, 2, 3 and 4 weeks. n=5. **b** PASMCs were isolated from control and rats induced by MCT for 1, 2, 3 and 4 weeks. n=7. **c** Total and phosphorylated CREB expression in hypoxia+su5416-induced PH. Lung tissues were isolated from control and mice induced by hypoxia+su5416 for 4 weeks. normoxia, n=5, hypoxia+su5416, n=6. *p<0.05 vs. Ctr.

**Additional file 2: Figure S2**. Total and phosphorylated CREB in hypoxia-treated PASMCs. **a** The levels of phosphorylated and total CREB in hypoxia-treated PASMCs. PASMCs were starved with serum-free DMEM/F12 for 24 h and then treated with hypoxia (2% O2) for 0min, 20min, 1h, 3h, 6h and 24h. n=12. **b** Serum-induced CREB phosphorylation and expression in PASMCs, PASMCs were starved with serum-free DMEM/F12 for 24 h and then treated with 10% fetal bovine serum (FBS) for 0min, 5min, 15min, 30min, 1h and 24h. n=4. **c** The levels of phosphorylated and total CREB in PASMCs treated with hypoxia. The PASMCs cultured in 10% FBS were exposed to 2% O2 for 0min, 20min, 1h, 3h, 6h and 24h. n=8. *p<0.05 vs. Ctr.

**Additional file 3: Figure S3**. Enhanced and prolonged serum-induced CREB phosphorylation in hypoxia-pretreated PASMCs. **a** Enhanced serum-induced CREB phosphorylation in hypoxia-pretreated PASMCs. The PASMCs were starved with serum-free DMEM/F12 and simultaneously exposed to hypoxia for 24h and then, the cells were stimulated by 10% fetal bovine serum (FBS) for 1 h in the present of hypoxia. n=3. **b** Prolonged serum-induced CREB phosphorylation in hypoxia-pretreated PASMCs. The PASMCs were starved with serum-free DMEM/F12 medium and simultaneously exposed to hypoxia for 24h, the cells were then stimulated by 10% fetal bovine serum for 1, 3, 6 and 24h in the present of hypoxia. n=5. Ctr, control; MCTW1, MCTW2, MCTW3 and MCTW4 represent MCT treatment for 1, 2, 3 and 4 weeks, respectively. *p<0.05 vs. Ctr, #p<0.05 vs. FBS 1h group.

**Additional file 4: Figure S4**. Identification of protein kinases involved in hypoxia-prolonged CREB phosphorylation. **a** The effect of ERK1/2, CAMK and PKC on CREB phosphorylation in hypoxia-treated PASMCs. Serum-starved PASMCs were pretreated with ERK1/2 inhibitor PD98059 (20 μM), CaMK inhibitor KN62 (10 μM) and PKC inhibitor chelerythrine chloride (CHE, 10 µM) for 24 h in the present of hypoxia, respectively. Then cells then were stimulated by 10% fetal bovine serum (FBS) for 1 h in the present of hypoxia and protein kinase inhibitors. n=5, 6 and 5. **b** The effect of P38MAPK, PI3K and PKA on CREB phosphorylation in hypoxia-treated PASMCs. Serum-starved PASMCs were pretreated with P38MAPK inhibitor SB203580 (20 μM), PI3K inhibitor LY294002 (50 μM) and PKA inhibitor H89 (10 μM) for 24h in the present of hypoxia, respectively. Then cells were stimulated by 10% FBS for 1 h in the present of hypoxia and protein kinase inhibitors. n=5, 5 and 5. **c** The effect of staurosporine (Sto, 10 nM) on CREB phosphorylation in hypoxia-pretreated PASMCs. The PASMCs were starved with serum-free DMEM/F12 and exposed to hypoxia for 24h, then, the cells were simultaneously stimulated by 10% FBS and Sto for 1 h in the present of hypoxia. n=6. *p<0.05 vs. Ctr, #p<0.05 vs. Hypoxia+10% FBS group.

**Additional file 5: Table S1**. The differentially expressed phosphatases in comparison of MCT-treatment 4 weeks with control.

**Additional file 6: Figure S5**. Profiling of phosphatases associated with CREB phosphorylation in hypoxia- and hypoxia+su5416-induced PH. Rows in the heatmap represent gene expression levels and columns represent each sample. The data were downloaded and extracted from gene expression omnibus repository with an accession number GSE8078.

**Additional file 7: Figure S6**. Plasmid profile of pGL4.20-TH and ZIP12 expression in MCT- and hypoxia+su5416-induced PH. **a** The TH promoter (-4490~+10) was linked to a luciferase reporter, pGL4.20 to make pGL4.20-TH (TH-promoter reporter). **b** Elevated expression of ZIP12 mRNA in rat lung tissues. n=6. **c** Elevated expression of ZIP12 mRNA in isolated PH-PASMCs, n= 5. *p<0.05 vs. Ctr. d Elevated expression of ZIP12 in lung tissues from hypoxia+su5416-induced PH model. n=4. *p<0.05 vs. hypoxia+su5416. TH, tyrosine hydroxylase.

## Data Availability

The datasets analysed during the current study are available in the gene expression omnibus repository with an accession number GSE149713, https://www.ncbi.nlm.nih.gov/geo/query/acc.cgi?acc=GSE149713. Other data generated or analysed during this study are included in this article and its supplementary information files.

## Abbreviations

PH: Pulmonary hypertension
CRE: CAMP response element
CREB: CAMP-response element binding protein
CREM: CAMP response element modulator
DEGs: Differentially expressed genes
DUSPs: Dual specificity phosphatases
FBS: Fetal bovine serum
MCT: Monocrotaline
MTT: Methyl thiazolyl tetrazolium bromide
PASMCs: Pulmonary artery smooth muscle cells
PH-PASMCs: PASMCs derived from rats with pulmonary hypertension
PCNA: Proliferating cell nuclear antigen
PP1: Protein phosphatase 1
PP2A: Protein phosphatase 2A
PTPs: Protein tyrosine phosphatases
RTKs: Receptor tyrosine kinases
TH: Tyrosine hydroxylase

## References

[1] Rabinovitch M (2012). Molecular pathogenesis of pulmonary arterial hypertension. J Clin Investig.

[2] Humbert M, Morrell NW, Archer SL, Stenmark KR, MacLean MR, Lang IM, Christman BW, Weir EK, Eickelberg O, Voelkel NF, Rabinovitch M: Cellular and molecular pathobiology of pulmonary arterial hypertension. J Am Coll Cardiol. 2004; 43(12 Suppl S)**:**13s–24s. 10.1016/j.jacc.2004.02.029 15194174

[3] Pilz RB, Casteel DE (2003). Regulation of gene expression by cyclic GMP. Circ Res.

[4] Ichiki T (2006). Role of cAMP response element binding protein in cardiovascular remodeling: Good, bad, or both?. Arterioscler Thromb Vasc Biol.

[5] Tokunou T, Shibata R, Kai H, Ichiki T, Morisaki T, Fukuyama K, Ono H, Iino N, Masuda S, Shimokawa H (2003). Apoptosis induced by inhibition of cyclic AMP response element-binding protein in vascular smooth muscle cells. Circulation.

[6] Ono H, Ichiki T, Fukuyama K, Iino N, Masuda S, Egashira K, Takeshita A (2004). cAMP-response element-binding protein mediates tumor necrosis factor-alpha-induced vascular smooth muscle cell migration. Arterioscler Thromb Vasc Biol.

[7] Leonard MO, Howell K, Madden SF, Costello CM, Higgins DG, Taylor CT, McLoughlin P (2008). Hypoxia selectively activates the CREB family of transcription factors in the in vivo lung. Am J Respir Crit Care Med.

[8] de Jesus DS, DeVallance E, Li Y, Falabella M, Guimaraes D, Shiva S, Kaufman BA, Gladwin MT, Pagano PJ (2019). Nox1/Ref-1-mediated activation of CREB promotes Gremlin1-driven endothelial cell proliferation and migration. Redox Biol.

[9] Song S, Ayon RJ, Yamamura A, Yamamura H, Dash S, Babicheva A, Tang H, Sun X, Cordery AG, Khalpey ZI (2016). Capsaicin-induced Ca2+ signaling is enhanced via upregulated TRPV1 channels in pulmonary artery smooth muscle cells from patients with idiopathic PAH. Am J Physiol Lung Cell Mol Physiol.

[10] Song S, Carr SG, McDermott KM, Rodriguez M, Babicheva A, Balistrieri A, Ayon RJ, Wang J, Makino A, Yuan JX (2018). STIM2 (stromal interaction molecule 2)-mediated increase in resting cytosolic free Ca(2+) concentration stimulates PASMC proliferation in pulmonary arterial hypertension. Hypertension.

[11] Ouyang S, Chen W, Gaofeng Z, Changcheng L, Guoping T, Minyan Z, Yang L, Min Y, Luo J (2021). Cyanidin-3-O-β-glucoside protects against pulmonary artery hypertension induced by monocrotaline via the TGF-β1/p38 MAPK/CREB signaling pathway. Mol Med Rep.

[12] Garat CV, Majka SM, Sullivan TM, Crossno JT, Reusch JEB, Klemm DJ (2020). CREB depletion in smooth muscle cells promotes medial thickening, adventitial fibrosis and elicits pulmonary hypertension. Pulm Circ.

[13] Jones C, Bisserier M, Bueno-Beti C, Bonnet G, Neves-Zaph S, Lee SY, Milara J, Dorfmüller P, Humbert M, Leopold JA (2020). A novel secreted-cAMP pathway inhibits pulmonary hypertension via a feed-forward mechanism. Cardiovasc Res.

[14] Zhuang W, Lian G, Huang B, Du A, Gong J, Xiao G, Xu C, Wang H, Xie L (2019). CPT1 regulates the proliferation of pulmonary artery smooth muscle cells through the AMPK-p53-p21 pathway in pulmonary arterial hypertension. Mol Cell Biochem.

[15] Xiao G, Wang T, Zhuang W, Ye C, Luo L, Wang H, Lian G, Xie L (2020). RNA sequencing analysis of monocrotaline-induced PAH reveals dysregulated chemokine and neuroactive ligand receptor pathways. Aging (Albany NY).

[16] Xiao G, Zhuang W, Wang T, Lian G, Luo L, Ye C, Wang H, Xie L (2020). Transcriptomic analysis identifies Toll-like and Nod-like pathways and necroptosis in pulmonary arterial hypertension. J Cell Mol Med.

[17] Zhao L, Oliver E, Maratou K, Atanur SS, Dubois OD, Cotroneo E, Chen CN, Wang L, Arce C, Chabosseau PL (2015). The zinc transporter ZIP12 regulates the pulmonary vascular response to chronic hypoxia. Nature.

[18] Lewis-Tuffin LJ, Quinn PG, Chikaraishi DM (2004). Tyrosine hydroxylase transcription depends primarily on cAMP response element activity, regardless of the type of inducing stimulus. Mol Cell Neurosci.

[19] Molina CA, Foulkes NS, Lalli E, Sassone-Corsi P (1993). Inducibility and negative autoregulation of CREM: an alternative promoter directs the expression of ICER, an early response repressor. Cell.

[20] Moreno-Vinasco L, Gomberg-Maitland M, Maitland ML, Desai AA, Singleton PA, Sammani S, Sam L, Liu Y, Husain AN, Lang RM (2008). Genomic assessment of a multikinase inhibitor, sorafenib, in a rodent model of pulmonary hypertension. Physiol Genomics.

[21] Zhang X, Odom DT, Koo SH, Conkright MD, Canettieri G, Best J, Chen H, Jenner R, Herbolsheimer E, Jacobsen E (2005). Genome-wide analysis of cAMP-response element binding protein occupancy, phosphorylation, and target gene activation in human tissues. Proc Natl Acad Sci USA.

[22] Kwapiszewska G, Wilhelm J, Wolff S, Laumanns I, Koenig IR, Ziegler A, Seeger W, Bohle RM, Weissmann N, Fink L (2005). Expression profiling of laser-microdissected intrapulmonary arteries in hypoxia-induced pulmonary hypertension. Respir Res.

[23] Shaywitz AJ, Greenberg ME (1999). CREB: a stimulus-induced transcription factor activated by a diverse array of extracellular signals. Annu Rev Biochem.

[24] Meggio F, Donella Deana A, Ruzzene M, Brunati AM, Cesaro L, Guerra B, Meyer T, Mett H, Fabbro D, Furet P, et al.: Different susceptibility of protein kinases to staurosporine inhibition. Kinetic studies and molecular bases for the resistance of protein kinase CK2. Eur J Biochem. 1995; 234(1)**:**317–22. 10.1111/j.1432-1033.1995.317_c.x 8529658

[25] Mayr BM, Canettieri G, Montminy MR (2001). Distinct effects of cAMP and mitogenic signals on CREB-binding protein recruitment impart specificity to target gene activation via CREB. Proc Natl Acad Sci USA.

[26] Kruger M, Schwaninger M, Blume R, Oetjen E, Knepel W (1997). Inhibition of CREB- and cAMP response element-mediated gene transcription by the immunosuppressive drugs cyclosporin A and FK506 in T cells. Naunyn Schmiedebergs Arch Pharmacol.

[27] Oetjen E, Grapentin D, Blume R, Seeger M, Krause D, Eggers A, Knepel W (2003). Regulation of human insulin gene transcription by the immunosuppressive drugs cyclosporin A and tacrolimus at concentrations that inhibit calcineurin activity and involving the transcription factor CREB. Naunyn Schmiedebergs Arch Pharmacol.

[28] Schwaninger M, Blume R, Oetjen E, Lux G, Knepel W (1993). Inhibition of cAMP-responsive element-mediated gene transcription by cyclosporin A and FK506 after membrane depolarization. J Biol Chem.

[29] Siemann G, Blume R, Grapentin D, Oetjen E, Schwaninger M, Knepel W (1999). Inhibition of cyclic AMP response element-binding protein/cyclic AMP response element-mediated transcription by the immunosuppressive drugs cyclosporin A and FK506 depends on the promoter context. Mol Pharmacol.

[30] Seidl MD, Steingraber AK, Wolf CT, Sur TM, Hildebrandt I, Witten A, Stoll M, Fischer JW, Schmitz W, Muller FU (2015). Transcription factor cAMP response element modulator (Crem) restrains Pdgf-dependent proliferation of vascular smooth muscle cells in mice. Pflugers Arch.

[31] Wilson M, Hogstrand C, Maret W (2012). Picomolar concentrations of free zinc(II) ions regulate receptor protein-tyrosine phosphatase beta activity. J Biol Chem.

[32] Haase H, Maret W (2005). Fluctuations of cellular, available zinc modulate insulin signaling via inhibition of protein tyrosine phosphatases. J Trace Elem Med Biol.

[33] Haase H, Maret W (2003). Intracellular zinc fluctuations modulate protein tyrosine phosphatase activity in insulin/insulin-like growth factor-1 signaling. Exp Cell Res.

[34] Wu W, Wang X, Zhang W, Reed W, Samet JM, Whang YE, Ghio AJ (2003). Zinc-induced PTEN protein degradation through the proteasome pathway in human airway epithelial cells. J Biol Chem.

[35] Ho Y, Samarasinghe R, Knoch ME, Lewis M, Aizenman E, DeFranco DB (2008). Selective inhibition of mitogen-activated protein kinase phosphatases by zinc accounts for extracellular signal-regulated kinase 1/2-dependent oxidative neuronal cell death. Mol Pharmacol.

[36] Xiong Y, Jing XP, Zhou XW, Wang XL, Yang Y, Sun XY, Qiu M, Cao FY, Lu YM, Liu R, Wang JZ (2013). Zinc induces protein phosphatase 2A inactivation and tau hyperphosphorylation through Src dependent PP2A (tyrosine 307) phosphorylation. Neurobiol Aging.

[37] Xiong Y, Luo DJ, Wang XL, Qiu M, Yang Y, Yan X, Wang JZ, Ye QF, Liu R (2015). Zinc binds to and directly inhibits protein phosphatase 2A in vitro. Neurosci Bull.

[38] Chowanadisai W, Graham DM, Keen CL, Rucker RB, Messerli MA (2013). Neurulation and neurite extension require the zinc transporter ZIP12 (slc39a12). Proc Natl Acad Sci USA.

[39] Rudolph D, Tafuri A, Gass P, Hammerling GJ, Arnold B, Schutz G (1998). Impaired fetal T cell development and perinatal lethality in mice lacking the cAMP response element binding protein. Proc Natl Acad Sci USA.

[40] Blendy JA, Kaestner KH, Schmid W, Gass P, Schutz G (1996). Targeting of the CREB gene leads to up-regulation of a novel CREB mRNA isoform. EMBO J.

[41] Li L, Howell K, Sands M, Banahan M, Frohlich S, Rowan SC, Neary R, Ryan D, McLoughlin P (2013). The alpha and Delta isoforms of CREB1 are required to maintain normal pulmonary vascular resistance. PLoS ONE.

[42] Carlezon WA, Duman RS, Nestler EJ (2005). The many faces of CREB. Trends Neurosci.

[43] Atsuta H, Uchiyama T, Kanai H, Iso T, Tanaka T, Suga T, Maeno T, Arai M, Nagai R, Kurabayashi M (2009). Effects of a stable prostacyclin analogue beraprost sodium on VEGF and PAI-1 gene expression in vascular smooth muscle cells. Int J Cardiol.

[44] Impey S, McCorkle SR, Cha-Molstad H, Dwyer JM, Yochum GS, Boss JM, McWeeney S, Dunn JJ, Mandel G, Goodman RH (2004). Defining the CREB regulon: a genome-wide analysis of transcription factor regulatory regions. Cell.

[45] Hartzell DD, Trinklein ND, Mendez J, Murphy N, Aldred SF, Wood K, Urh M (2009). A functional analysis of the CREB signaling pathway using HaloCHIP-chip and high throughput reporter assays. BMC Genom.

[46] Beitner-Johnson D, Millhorn DE (1998). Hypoxia induces phosphorylation of the cyclic AMP response element-binding protein by a novel signaling mechanism. J Biol Chem.

[47] Wadzinski BE, Wheat WH, Jaspers S, Peruski LF, Lickteig RL, Johnson GL, Klemm DJ (1993). Nuclear protein phosphatase 2A dephosphorylates protein kinase A-phosphorylated CREB and regulates CREB transcriptional stimulation. Mol Cell Biol.

[48] Perrotti D, Neviani P (2013). Protein phosphatase 2A: a target for anticancer therapy. Lancet Oncol.

[49] Sankhe S, Manousakidi S, Antigny F, Ataam JA, Bentebbal S, Ruchon Y, Lecerf F, Sabourin J, Price L, Fadel E (2017). T-type Ca2+ channels elicit pro-proliferative and anti-apoptotic responses through impaired PP2A/Akt1 signaling in PASMCs from patients with pulmonary arterial hypertension. Biochim Biophys Acta.

[50] Hagiwara M, Alberts A, Brindle P, Meinkoth J, Feramisco J, Deng T, Karin M, Shenolikar S, Montminy M (1992). Transcriptional attenuation following cAMP induction requires PP-1-mediated dephosphorylation of CREB. Cell.

[51] ten Freyhaus H, Dagnell M, Leuchs M, Vantler M, Berghausen EM, Caglayan E, Weissmann N, Dahal BK, Schermuly RT, Ostman A (2011). Hypoxia enhances platelet-derived growth factor signaling in the pulmonary vasculature by down-regulation of protein tyrosine phosphatases. Am J Respir Crit Care Med.

[52] Vahidi Ferdowsi P, Ng R, Adulcikas J, Sohal SS, Myers S (2020). Zinc modulates several transcription-factor regulated pathways in mouse skeletal muscle cells. Molecules.

[53] Bernal PJ, Bauer EM, Cao R, Maniar S, Mosher M, Chen J, Wang QJ, Glorioso JC, Pitt BR, Watkins SC, St Croix CM (2011). A role for zinc in regulating hypoxia-induced contractile events in pulmonary endothelium. Am J Physiol Lung Cell Mol Physiol.

[54] Bernal PJ, Leelavanichkul K, Bauer E, Cao R, Wilson A, Wasserloos KJ, Watkins SC, Pitt BR, St Croix CM (2008). Nitric-oxide-mediated zinc release contributes to hypoxic regulation of pulmonary vascular tone. Circ Res.

[55] Tran HB, Maiolo S, Harper R, Zalewski PD, Reynolds P, Hodge S: Dysregulated zinc and sphingosine-1-phosphate signalling in pulmonary hypertension: potential effects by targeting of bone morphogenetic protein receptor type 2 in pulmonary microvessels. Cell Biol Int. 2021. 10.1002/cbin.11682 34347342

