# Supplementary figures and images for: Zinc-mediated activation of CREB pathway in proliferation of pulmonary artery smooth muscle cells in pulmonary hypertension
Source: Cell Commun Signal. 2021 Oct 11;19:103. doi: 10.1186/s12964-021-00779-y (PMC8504081; doi:10.1186/s12964-021-00779-y)

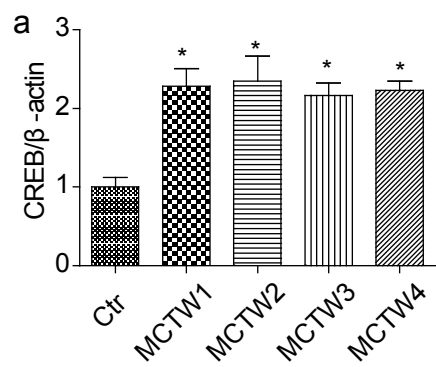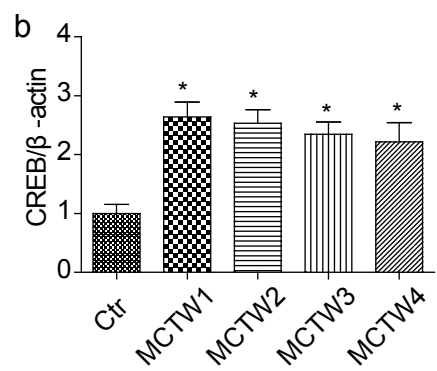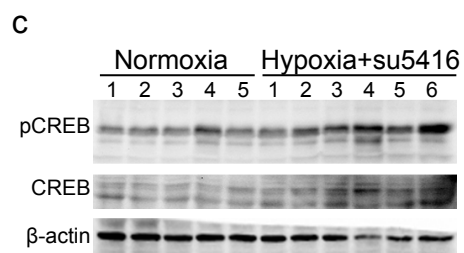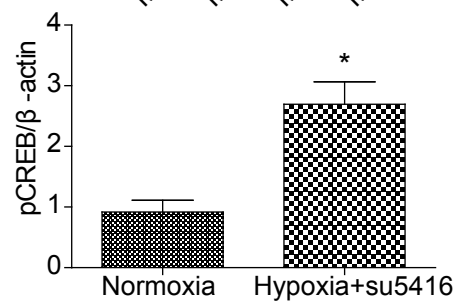

Supplement: Supplementary file 2 — Additional file 1: Figure S1. Total and phosphorylated CREB expression in lung and PASMCs. a Lung tissues were isolated from control and rats induced by MCT for 1, 2, 3 and 4 weeks. n=5. b PASMCs were isolated from control and rats induced by MCT for 1, 2, 3 and 4 weeks. n=7. c Total and phosphorylated CREB expression in hypoxia+su5416-induced PH. Lung tissues were isolated from control and mice induced by hypoxia+su5416 for 4 weeks. normoxia, n=5, hypoxia+su5416, n=6. *p<0.05 vs. Ctr. [file 12964_2021_779_MOESM2_ESM.pdf]

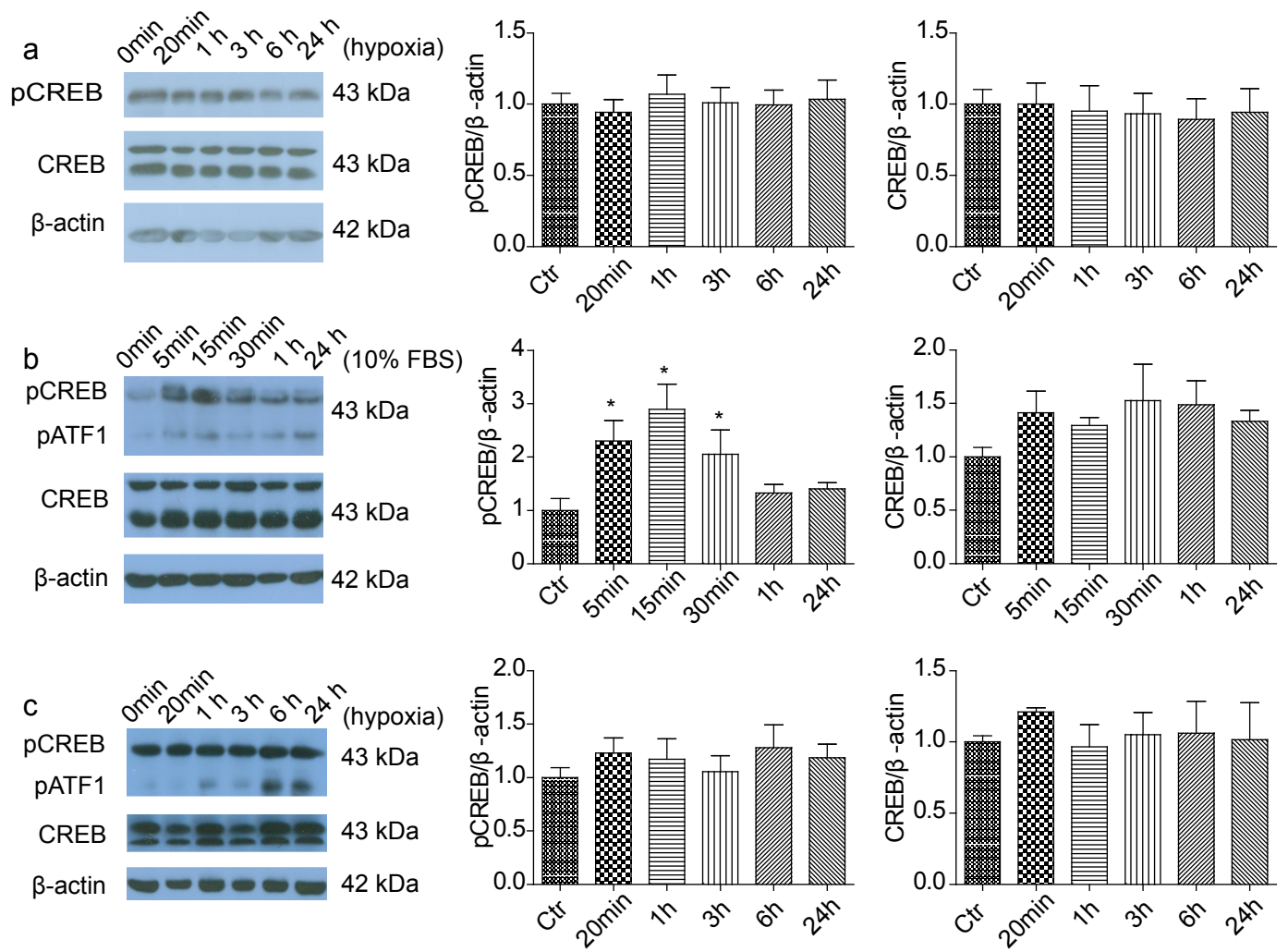

Supplement: Supplementary file 3 — Additional file 2: Figure S2. Total and phosphorylated CREB in hypoxia-treated PASMCs. a The levels of phosphorylated and total CREB in hypoxia-treated PASMCs. PASMCs were starved with serum-free DMEM/F12 for 24 h and then treated with hypoxia (2% O2) for 0min, 20min, 1h, 3h, 6h and 24h. n=12. b Serum-induced CREB phosphorylation and expression in PASMCs, PASMCs were starved with serum-free DMEM/F12 for 24 h and then treated with 10% fetal bovine serum (FBS) for 0min, 5min, 15min, 30min, 1h and 24h. n=4. c The levels of phosphorylated and total CREB in PASMCs treated with hypoxia. The PASMCs cultured in 10% FBS were exposed to 2% O2 for 0min, 20min, 1h, 3h, 6h and 24h. n=8. *p<0.05 vs. Ctr. [file 12964_2021_779_MOESM3_ESM.pdf]

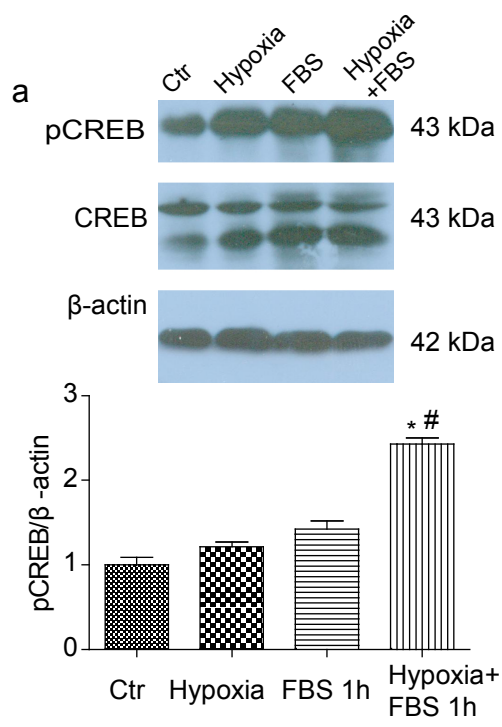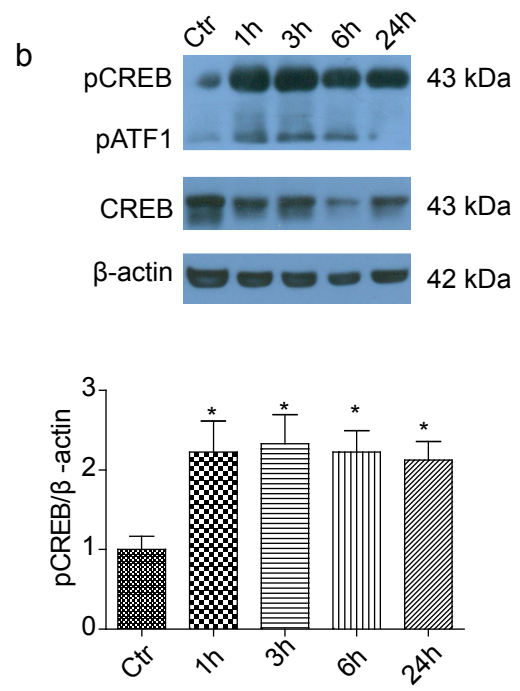

Supplement: Supplementary file 4 — Additional file 3: Figure S3. Enhanced and prolonged serum-induced CREB phosphorylation in hypoxia-pretreated PASMCs. a Enhanced serum-induced CREB phosphorylation in hypoxia-pretreated PASMCs. The PASMCs were starved with serum-free DMEM/F12 and simultaneously exposed to hypoxia for 24h and then, the cells were stimulated by 10% fetal bovine serum (FBS) for 1 h in the present of hypoxia. n=3. b Prolonged serum-induced CREB phosphorylation in hypoxia-pretreated PASMCs. The PASMCs were starved with serum-free DMEM/F12 medium and simultaneously exposed to hypoxia for 24h, the cells were then stimulated by 10% fetal bovine serum for 1, 3, 6 and 24h in the present of hypoxia. n=5. Ctr, control; MCTW1, MCTW2, MCTW3 and MCTW4 represent MCT treatment for 1, 2, 3 and 4 weeks, respectively. *p<0.05 vs. Ctr, #p<0.05 vs. FBS 1h group. [file 12964_2021_779_MOESM4_ESM.pdf]

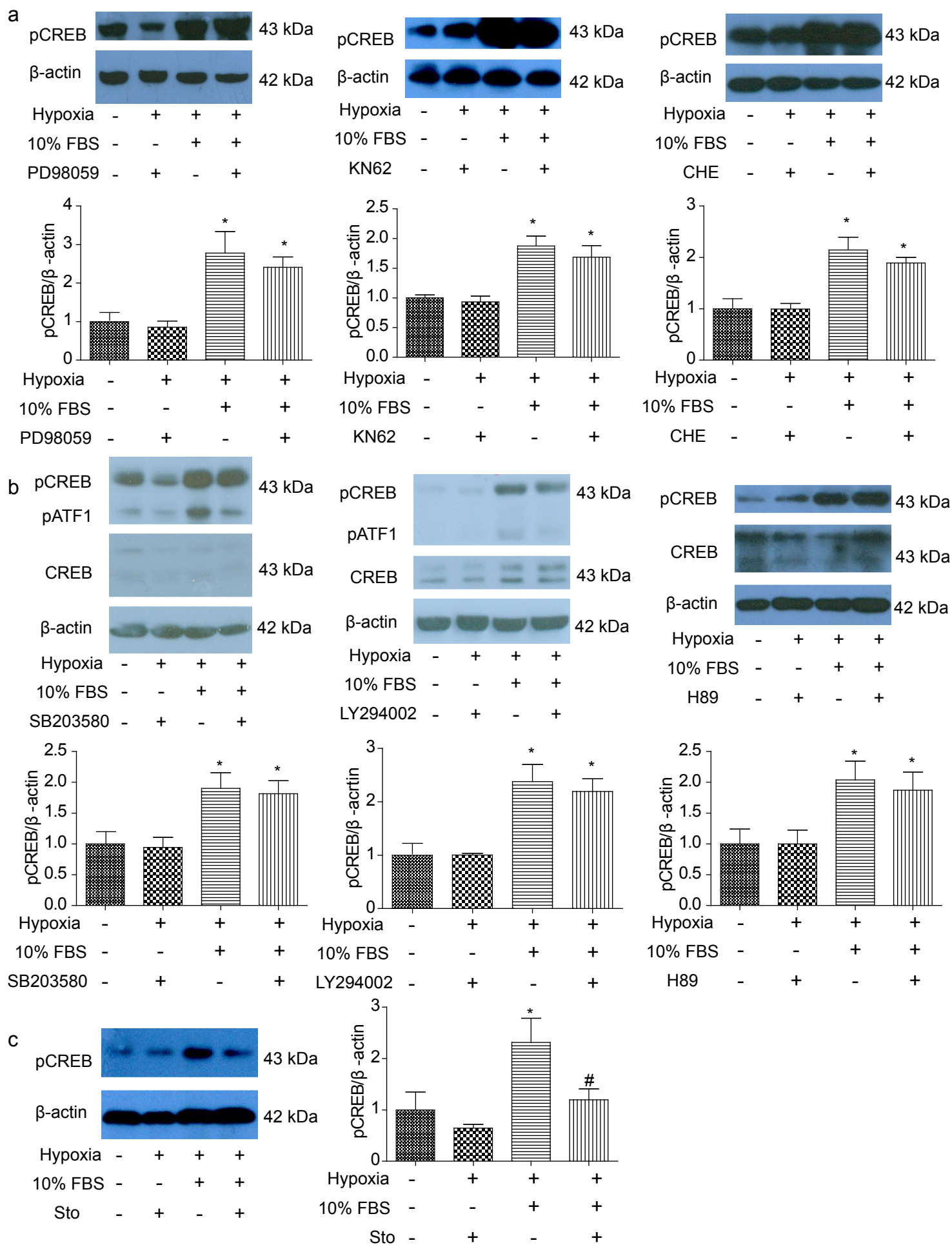

Supplement: Supplementary file 5 — Additional file 4: Figure S4. Identification of protein kinases involved in hypoxia-prolonged CREB phosphorylation. a The effect of ERK1/2, CAMK and PKC on CREB phosphorylation in hypoxia-treated PASMCs. Serum-starved PASMCs were pretreated with ERK1/2 inhibitor PD98059 (20 μM), CaMK inhibitor KN62 (10 μM) and PKC inhibitor chelerythrine chloride (CHE, 10 µM) for 24 h in the present of hypoxia, respectively. Then cells then were stimulated by 10% fetal bovine serum (FBS) for 1 h in the present of hypoxia and protein kinase inhibitors. n=5, 6 and 5. b The effect of P38MAPK, PI3K and PKA on CREB phosphorylation in hypoxia-treated PASMCs. Serum-starved PASMCs were pretreated with P38MAPK inhibitor SB203580 (20 μM), PI3K inhibitor LY294002 (50 μM) and PKA inhibitor H89 (10 μM) for 24h in the present of hypoxia, respectively. Then cells were stimulated by 10% FBS for 1 h in the present of hypoxia and protein kinase inhibitors. n=5, 5 and 5. c The effect of staurosporine (Sto, 10 nM) on CREB phosphorylation in hypoxia-pretreated PASMCs. The PASMCs were starved with serum-free DMEM/F12 and exposed to hypoxia for 24h, then, the cells were simultaneously stimulated by 10% FBS and Sto for 1 h in the present of hypoxia. n=6. *p<0.05 vs. Ctr, #p<0.05 vs. Hypoxia+10% FBS group. [file 12964_2021_779_MOESM5_ESM.pdf]

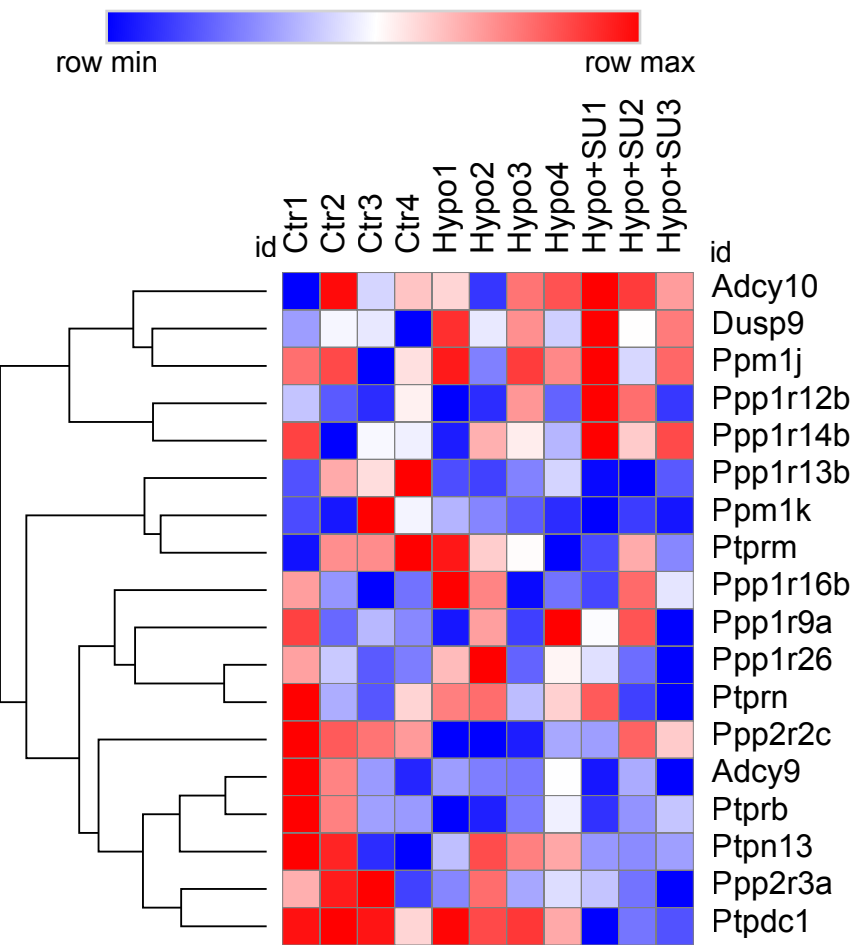

Supplement: Supplementary file 7 — Additional file 6: Figure S5. Profiling of phosphatases associated with CREB phosphorylation in hypoxia- and hypoxia+su5416-induced PH. Rows in the heatmap represent gene expression levels and columns represent each sample. The data were downloaded and extracted from gene expression omnibus repository with an accession number GSE8078. [file 12964_2021_779_MOESM7_ESM.pdf]

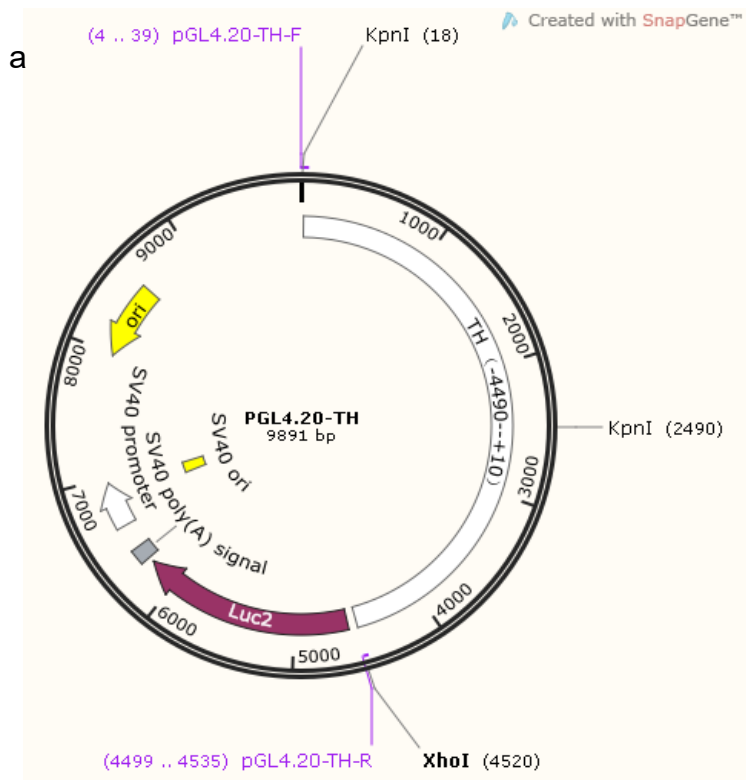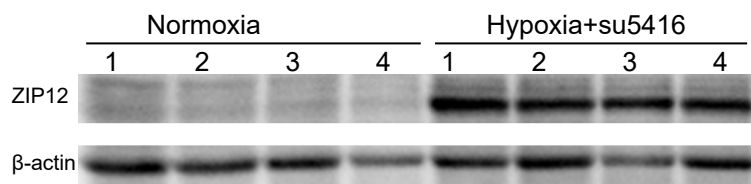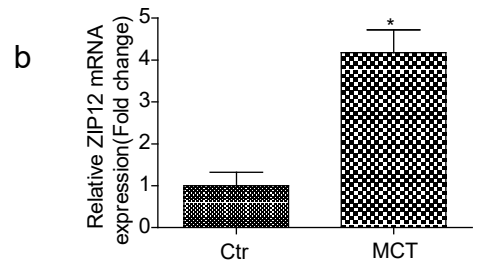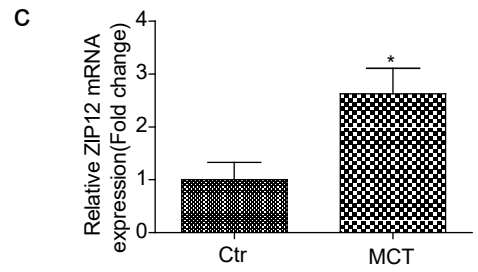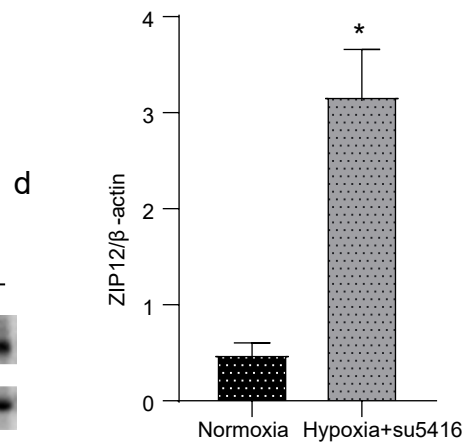

Supplement: Supplementary file 8 — Additional file 7: Figure S6. Plasmid profile of pGL4.20-TH and ZIP12 expression in MCT- and hypoxia+su5416-induced PH. a The TH promoter (-4490~+10) was linked to a luciferase reporter, pGL4.20 to make pGL4.20-TH (TH-promoter reporter). b Elevated expression of ZIP12 mRNA in rat lung tissues. n=6. c Elevated expression of ZIP12 mRNA in isolated PH-PASMCs, n= 5. *p<0.05 vs. Ctr. d Elevated expression of ZIP12 in lung tissues from hypoxia+su5416-induced PH model. n=4. *p<0.05 vs. hypoxia+su5416. TH, tyrosine hydroxylase. [file 12964_2021_779_MOESM8_ESM.pdf]
